# The extracellular vesicle proteomes of *Sorghum bicolor* and *Arabidopsis thaliana* are partially conserved

**DOI:** 10.1093/plphys/kiad644

**Published:** 2023-12-04

**Authors:** Timothy Chaya, Aparajita Banerjee, Brian D Rutter, Deji Adekanye, Jean Ross, Guobin Hu, Roger W Innes, Jeffrey L Caplan

**Affiliations:** Department of Plant and Soil Sciences, University of Delaware, Newark, DE 19716, USA; Delaware Biotechnology Institute, University of Delaware, Newark, DE 19716, USA; Department of Plant and Soil Sciences, University of Delaware, Newark, DE 19716, USA; Delaware Biotechnology Institute, University of Delaware, Newark, DE 19716, USA; Department of Biology, Indiana University, Bloomington, IN 47405, USA; Delaware Biotechnology Institute, University of Delaware, Newark, DE 19716, USA; Department of Biological Sciences, University of Delaware, Newark, DE 19716, USA; Delaware Biotechnology Institute, University of Delaware, Newark, DE 19716, USA; The Laboratory for Biomolecular Structures, Brookhaven National Laboratory, Upton, NY 11973, USA; Department of Biology, Indiana University, Bloomington, IN 47405, USA; Department of Plant and Soil Sciences, University of Delaware, Newark, DE 19716, USA; Delaware Biotechnology Institute, University of Delaware, Newark, DE 19716, USA; Department of Biological Sciences, University of Delaware, Newark, DE 19716, USA

## Abstract

Plant extracellular vesicles (EVs) are membrane-bound organelles involved mainly in intercellular communications and defense responses against pathogens. Recent studies have demonstrated the presence of proteins, nucleic acids including small RNAs, and lipids along with other metabolites in plant EVs. Here, we describe the isolation and characterization of EVs from sorghum (*Sorghum bicolor*). Nanoparticle tracking analysis, dynamic light scattering, and cryo-electron tomography showed the presence of a heterogeneous population of EVs isolated from the apoplastic wash of sorghum leaves. Cryo-electron microscopy revealed that EVs had a median size of 110 nm and distinct populations of vesicles with single or multiple lipid bilayers and low or high amounts of contents. The heterogeneity was further supported by data showing that only a subset of EVs that were stained with a membrane dye, Potomac Gold, were also stained with the membrane-permeant esterase-dependent dye, calcein acetoxymethyl ester. Proteomic analysis identified 437 proteins that were enriched in multiple EV isolations, with the majority of these also found in the EV proteome of Arabidopsis (*Arabidopsis thaliana*). These data suggest a partial conservation of EV contents and function between the monocot, sorghum, and a distantly related eudicot, Arabidopsis.

## Teaser

Extracellular vesicles (EVs) isolated from sorghum have a heterogeneous morphology and contain proteins also found in isolated Arabidopsis EVs, including the commonly used marker PENETRATION1.

## Introduction

Extracellular vesicles (EVs) are lipid bilayer membrane-enclosed nanoparticles released by the cells of various organisms ranging from prokaryotes to eukaryotes into extracellular spaces (Yáñez-Mó et al. 2015; Tkach and Théry 2016; Cui et al. 2019; LeClaire et al. 2020). EVs mediate intercellular communications, both proximal and distal to the place of origin, through the delivery of proteins, nucleic acids including small RNAs, lipids, and other metabolites (Yáñez-Mó et al. 2015; Tkach and Théry 2016; Maas et al. 2017; Wu et al. 2019; Woith et al. 2021; Liu et al. 2022). Previously, EVs were classified into 3 main categories: apoptotic bodies (1,000 to 5,000 nm in diameter) generated during programmed cell death via cell fragmentation and blebbing of plasma membrane, microvesicles (100 to 1,000 nm in diameter) released by direct budding and shedding of plasma membrane, and exosomes (30 to 150 nm in diameter) originated intracellularly as multivesicular bodies (MVBs) that fuse with the plasma membrane (PM) to release intraluminal vesicles outside the cell (György et al. 2011; Théry 2011; van der Pol et al. 2012; Akers et al. 2013; Raposo and Stoorvogel 2013; Lawson et al. 2016; Rutter and Innes 2017; Zempleni et al. 2017; Woith et al. 2019). However, lacking clear evidence of biogenesis or biomarkers, it is necessary to categorize EVs with physical characteristics such as buoyancy, molecular cargo, or size (Théry et al. 2018). In the field of EV research, mammalian EVs are widely studied. They are released from almost all cell types and body fluids and play critical roles in immune responses and the progression of various diseases (Colombo et al. 2014; Woith et al. 2019). Based on their importance as carriers of biomolecules as well as potential vehicles equipped to cross challenging pharmacological barriers, mammalian EVs have been extensively explored in clinical and therapeutic applications (van der Pol et al. 2012; Elsharkasy et al. 2020).

Compared to the comprehensive research encompassing mammalian EVs, the research in the field of plant EVs is still in its infancy. Plant EVs were discovered in the 1960s, where they were described in carrot (*Daucus carota*) cell culture and in wheat (*Triticum aestivum*; Manocha and Shaw 1964; Halperin and Jensen 1967). The current evidence suggests plant EVs play a role in intercellular and extracellular communications, cross-kingdom exchange of small RNAs, plant immune responses, and plant–microbe symbiosis (Regente et al. 2017; Rutter and Innes 2017; 2018; Cai et al. 2018, 2019; Rutter and Innes 2020; Liu et al. 2021; Wang et al. 2023). Despite these findings, there remain many fundamental questions regarding plant EVs and their potential applications, even 6 decades after their discovery (Urzi et al. 2021). Further research needs to be done to shed light on their biogenesis, morphology, packaging of cargoes, mechanism of passage through the cell wall, interkingdom movement, and EV cargo delivery to name a few.

Various biofluids including blood, urine, cerebrospinal fluid, lymphatics, tears, saliva and nasal secretions, ascites, and semen have been used as sources of mammalian EVs (Akers et al. 2013). Stepwise differential centrifugation is used most often to isolate EVs from mammalian biofluids. Briefly, fluids are processed at a low centrifugation speed to remove cellular debris and large particles followed by ultracentrifugation at a higher speed, typically at 100,000 *× g*, to pellet EVs (Théry et al. 2006; Akers et al. 2013; Gardiner et al. 2016). Further purification can be obtained by ultracentrifugation of the crude EVs using a discontinuous density gradient (Raposo et al. 1996; Akers et al. 2013).

In the plant sciences, EVs are most frequently isolated from apoplastic wash fluids. Such fluids are obtained by vacuum infiltrating a buffer into leaves and using slow centrifugation speeds to push the buffer out of the leaves through stomatal openings (Rutter and Innes 2017; Liu, Wang, et al. 2020; Schlemmer et al. 2021). This process effectively rinses the extracellular spaces inside the leaves and allows researchers to collect any molecules or particles therein. To date, EVs from the wash fluids of Arabidopsis (*Arabidopsis thaliana*) and barley (*Hordeum vulgare*) leaves have been isolated using differential ultracentrifugation. Additionally, many studies have isolated plant-derived edible nanoparticles (PDNPs) or plant-derived vesicles (PDVs) from the juices of various fruits and vegetables, such as grapefruit (*Citrus* × *paradisi*), grape (*Vitis vinifera*), lemon (*Citrus limon*), strawberry (*Fragaria* × *ananassa*), orange (*Citrus* × *sinensis*), ginseng (*Panax ginseng*), ginger (*Zingiber officinale*), carrot, broccoli (*Brassica oleracea* var. *italica*), and cabbage (*B. oleracea* var. *capitata*; Wang et al. 2013; Mu et al. 2014; Raimondo et al. 2015; Zhang et al. 2016; Deng et al. 2017; Yang et al. 2018; Cao et al. 2019; Iravani and Varma 2019; Berger et al. 2020; Yang et al. 2020; Man et al. 2021; Perut et al. 2021; Savcı et al. 2021; You et al. 2021). PDVs have potential therapeutic and clinical applications, but since they are often a mixture of vesicles with intracellular and extracellular origins, PDVs should not be used to study the natural formation and function of EVs.

While there is an understanding that these vesicles play a key role in the response to various pathogens, particularly fungi, defining their function in the immune response is an ongoing process. It is also unknown how conserved are the cargo and composition of plant EVs from monocots to eudicots.

To expand our knowledge of plant EVs, we describe here methods for isolating and purifying EVs from the important cereal crop sorghum (*Sorghum bicolor*). EVs from this monocot were isolated from the wash fluids of leaves and were similar both in morphology and protein content to EVs from Arabidopsis. In this study, we described an optimized protocol for EV isolation from sorghum leaves using differential centrifugation methods. We characterized the isolated sorghum EVs using nanoparticle tracking analysis (NTA), dynamic light scattering (DLS) analysis, light and electron microscopy, and MS to identify the sorghum EV proteome.

## Results

### Isolation of sorghum EVs

**Figure 1.**
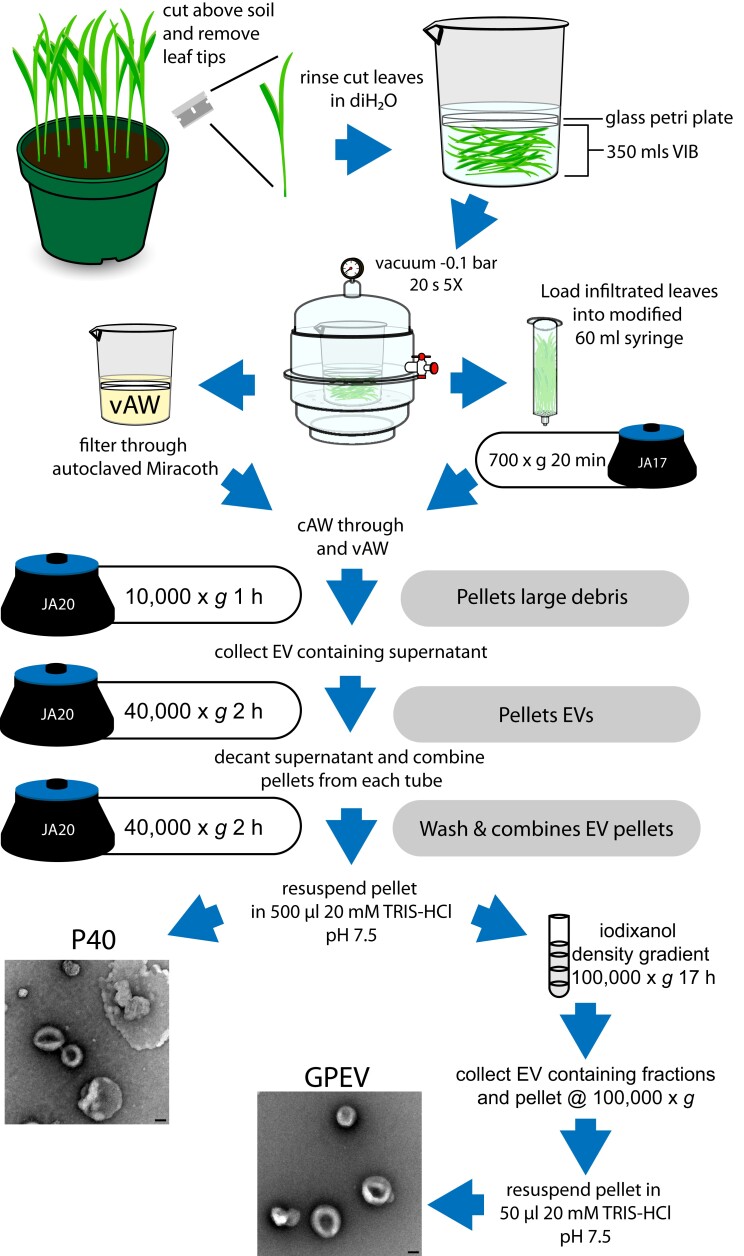
Sorghum EV isolation protocol. Sorghum leaves were vacuum infiltrated with VIB, and EVs were isolated from the vAW and the cAW to generate the resuspended pellet from the 40,000 × *g* spin (P40) EV fraction. EVs were further purified on an iodixanol gradient to generate a GPEV fraction. In all images, the scale bar in TEM images equals 50 nm.

We used the differential centrifugation method to isolate EVs from sorghum plants by adapting the method described by Rutter and Innes (2017; Fig. 1). Briefly, approximately 14-d-old sorghum plants were cut above the soil and vacuum infiltrated in vesicle isolation buffer (VIB). The centrifugation-based apoplastic wash (cAW) was collected from the infiltrated plants at a relative centrifugal force (RCF) of 700 × *g*. Initially, we continued with only the cAW for the downstream differential centrifugation steps. However, the low yield of EVs from our multiple trials prompted us to check if EVs were getting released into the VIB by the repetitive changes in vacuum pressure. To look for EV-like particles in the residual VIB from vacuum treatments (vacuum-based apoplastic wash [vAW]), we stained the vAW with the membrane dye Potomac Gold. Spinning disk confocal microscopy detected EV-like objects in vAW (Supplemental Fig. S1). Therefore, we used the combined cAW and vAW for the subsequent downstream centrifugation steps. The P40 sorghum EVs were obtained by centrifuging the combined cAW and vAW initially at 10,000 × *g* to remove the large cellular debris followed by 2 rounds of subsequent centrifugation at 39,800 × *g* (Fig. 1). Further purification was accomplished using a discontinuous iodixanol (OptiPrep) gradient (Rutter and Innes 2017). Briefly, the P40 pellet was loaded onto the top of the iodixanol gradient and centrifuged for 17 h at 100,000 × *g*. The fractions spanning the interface between the 10% and 20% iodixanol layers were collected and centrifuged again at 100,000 × *g* for 1 h to obtain gradient-purified EVs (GPEVs).

### Characterization of sorghum EV size

**Figure 2.**
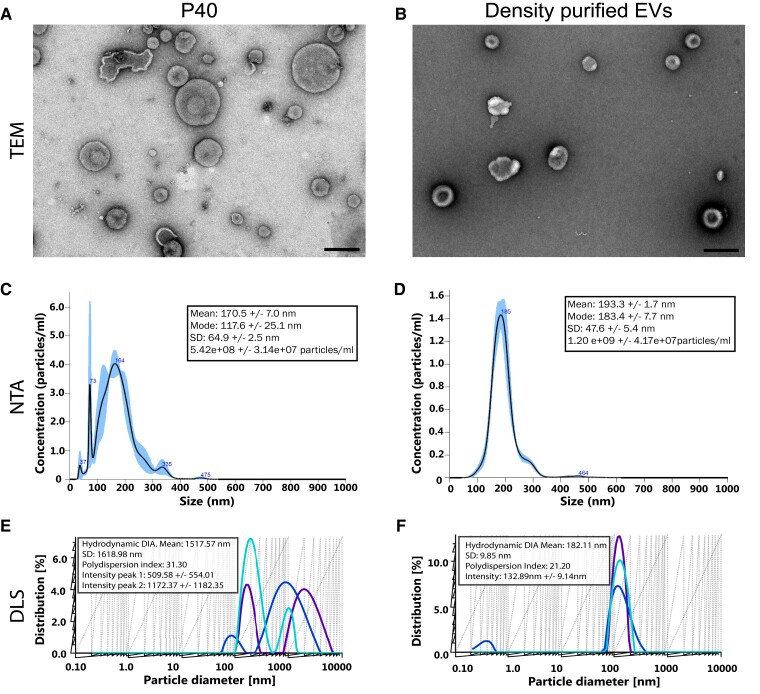
Characterization of P40 and GPEVs. **A, B)** P40 **A)** and iodixanol density-purified EVs **B)** were negatively stained and imaged by TEM. Scale bar, 200 nm in all images. **C, D)** NTA of EVs. Shaded area represents the Se of the mean of 3 technical replicates. **E, F)** DLS of EVs. Colors represent technical replicates.

We first analyzed the morphology of the EV-like particles obtained from sorghum leaves by negative-stain transmission electron microscopy (TEM). The EV isolation was divided into 2 portions, the P40 containing pellet and GPEVs. EVs from both fractions appeared as numerous cup-shaped objects of various sizes in TEM micrographs (Fig. 2, A and B). The appearance of our EVs as cup shaped is similar to TEM images of EVs obtained from Arabidopsis leaves and PDV isolated from strawberry, lemon, orange, and cabbage juices (Rutter and Innes 2017; Baldini et al. 2018; Berger et al. 2020; Yang et al. 2020; Liu, Wu, et al. 2020; Perut et al. 2021; You et al. 2021). TEM images of the P40 fraction revealed a heterogeneous size distribution of EV-like particles. In contrast, the GPEVs were more homogeneously sized.

We used NTA to determine the size and yield of the particles in the P40 pellet. The P40 EVs had a mean size of 170.5 nm, and GPEVs had a mean size of 193.3 nm (Fig. 2, C and D). The observation of multiple peaks and large Sd in the NTA analysis indicates a heterogeneous population of EV particles of varying sizes. Similar size distribution was reported for EV-like particles from Arabidopsis leaves that were obtained from the P40 (Rutter and Innes 2017). They demonstrated that the particle size of the P40, isolated from the Arabidopsis leaves using similar techniques as described in our study, ranged approximately from 50 to 300 nm in diameter and particles around 150 nm in diameter were the predominant species (Rutter and Innes 2017). NTA has a detection size limit of 1,000 nm, and therefore, DLS was used to detect larger vesicles. DLS of P40 EVs measured their hydrodynamic radius mean as 1,517.6 nm with a Sd of 1,619 nm and 2 peaks at 160.88 nm and 1,521.07 nm (Fig. 2, E and F). A large amount of variation was observed in the peaks and percent distribution of the technical replicates. In contrast, the DLS of GPEVs had a single peak with a mean value of 182.23 nm with a relatively low Sd of 9.85 nm, suggesting the EV size was more homogeneous. These differences in size distribution seen by the NTA and DLS measurements are in agreement with the TEM analysis.

### Characterization of morphology

**Figure 3.**
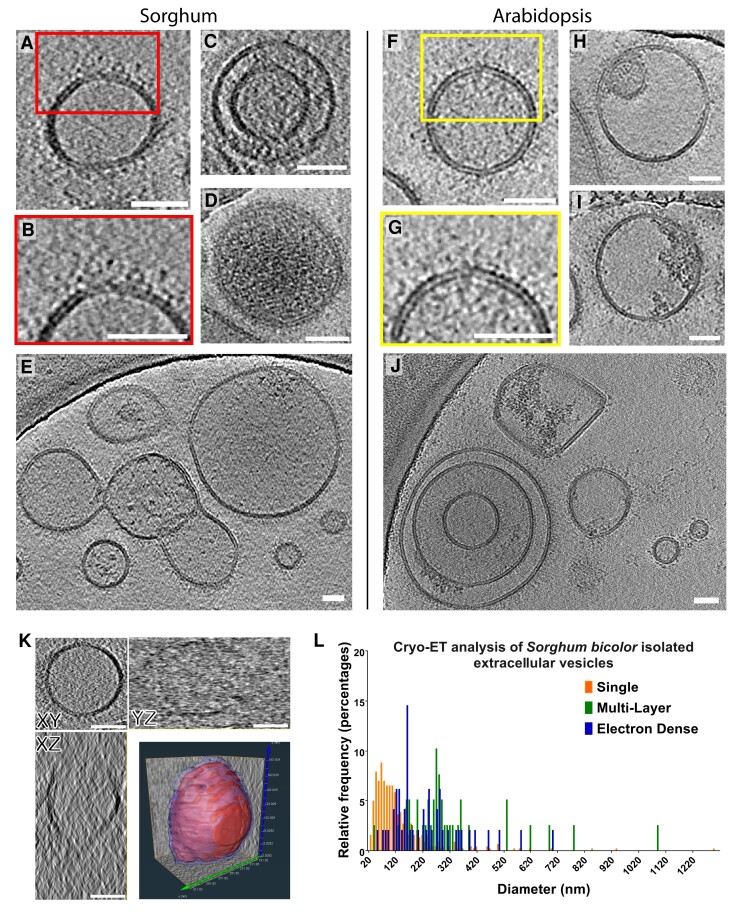
Analysis of cryo-ET sorghum and Arabidopsis P40 EVs. **A to E)** Sorghum EVs. **A)** A single vesicle showing the lipid bilayer. **B)** Enlarged area of the vesicle showing electron-dense material associated on the external surface. **C)** Multilayer EV. **D)** Electron-dense EV. **E)** Field showing the mixed variety of EVs seen in the P40 pellet. **F to I)** Arabidopsis EVs. **F)** A single vesicle with visible lipid bilayer. **G)** Enlarged image showing proteins associated with external surface. **H)** Multilayer EV. **I)** Electron-dense material associated with the outside of the EV. **J)** Variation of vesicles seen in P40 pellet. **K)** Single-layer sorghum vesicle rendered in Amira. The solid red core shows the inner boundary of the vesicle, and the translucent light blue shows the fringes on the vesicle. **L)** A histogram of the measured sorghum vesicles split into 3 categories. The mean size is 150.95 nm and a median of 110.00 nm. Scale bar equals 50 nm in all images.

To further characterize the EVs, we performed cryo-electron tomography (cryo-ET). The P40 fraction with greater variation was chosen for cryo-ET to obtain a more complete analysis of types of EV-like particles that can be purified from sorghum. Cryo-ET of EVs from Arabidopsis was conducted as a comparison. Both sorghum and Arabidopsis EVs were a mixture of vesicles with a single lipid bilayer (Fig. 3, A, B, D to G, I, and J) or multiple lipid bilayers (Fig. 3, C, H, and J; Supplemental Fig. S2). The multilayered EVs had more than 1 lipid bilayer with the most common being 2 bilayers as seen in Fig. 3, C and H. Electron-dense material was observed inside and associated with the surface of sorghum (Fig. 3D) and Arabidopsis (Fig. 3I) EVs. Both single and multilayered EVs contained electron-dense material, and a difference in density between the different layers of multilayered EVs was observed in sorghum (Supplemental Fig. S2) and Arabidopsis (Fig. 3J). Another prominent characteristic of EVs from both sorghum and Arabidopsis was an electron-dense coating or protruding structures from the surface (Fig. 3, A, B, F, and G). 3D rendering of a single vesicle showed that they were relatively spherical in structure, and it was possible to separate the electron-dense external coating from the more defined lipid bilayer (Fig. 3K). A 3D rendering of a single vesicle showed that they were relatively spherical in structure, and it was possible to separate the fringed edge from the more defined lipid bilayer (Fig. 3K). Next, we did a more extensive analysis of sorghum EVs, the focus of this study. The cryo-ET image analysis of 483 EVs showed that 92% of the sorghum EVs were single layered (444) and only 6.8% were multilayered (39) EVs (Fig. 3L). The single layered EVs were smaller and had a mean diameter of 135.58 nm and a median diameter of 104.03 nm with a Se of 5.78 nm. The multilayered EVs were larger and had a mean diameter of 322.31 nm and a median diameter of 274.67 nm with a Se of 31.24 nm. We also quantitated the number of EVs that appeared electron dense in the cryo-ET micrographs. Eight percent of the sorghum EVs (43) were electron dense, which suggests these have a higher concentration of cargo (Fig. 3L).

### Confocal microscopy of sorghum EVs

**Figure 4.**
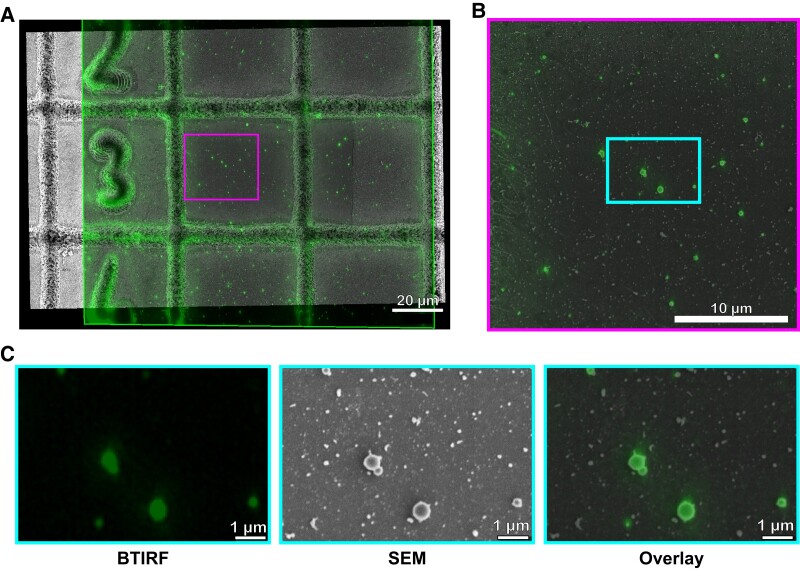
Correlative image analysis. **A)** TIRF image of the calcein AM–stained sorghum vesicles (green) overlaid on a corresponding SEM image (grayscale). Scale bar equals 20 *µ*m. **B)** A magnified view of the boxed area in **A)**. Scale bar equals 10 *µ*m. **C)** A magnified view of the boxed area in **B)** showing the individual TIRF (left), SEM (center), and correlative overlay of TIRF and SEM (right). Scale bars equal 1 *µ*m.

To characterize sorghum EVs using light microscopy approaches, we developed a method that uses both a soluble and lipophilic membrane fluorescent dye. First, we explored the soluble, membrane-permeable fluorescent dye, calcein acetoxymethyl ester (AM), for labeling isolated sorghum EVs. Calcein AM, the acetomethoxy derivative of calcein, is commonly used for cell viability assay and has been used previously to label EVs (Hiraoka and Kimbara 2002; Bauer et al. 2003; Bratosin et al. 2005; Gray et al. 2015; Miles et al. 2015). Calcein AM green is a nonfluorescent esterase-dependent substrate and is lipophilic in nature, which allows it to passively cross membranes (Bratosin et al. 2005). Once inside EVs, calcein AM is hydrolyzed to become fluorescent and membrane impermeant, trapping the dye inside EVs with an intact lipid bilayer. After incubating the P40 sorghum EVs with calcein AM green, we observed small fluorescent foci using spinning disk confocal microscopy (Fig. 4). To determine if these spots corresponded to EVs, we conducted correlative light and electron microscopy (CLEM; Fig. 4). EVs were deposited on gridded coverglasses and imaged using the Dragonfly spinning disk and subsequently by scanning electron microscopy (SEM). One hundred twenty-two stained vesicles corresponded to round vesicles of the expected size range between the correlated confocal and SEM images.

**Figure 5.**
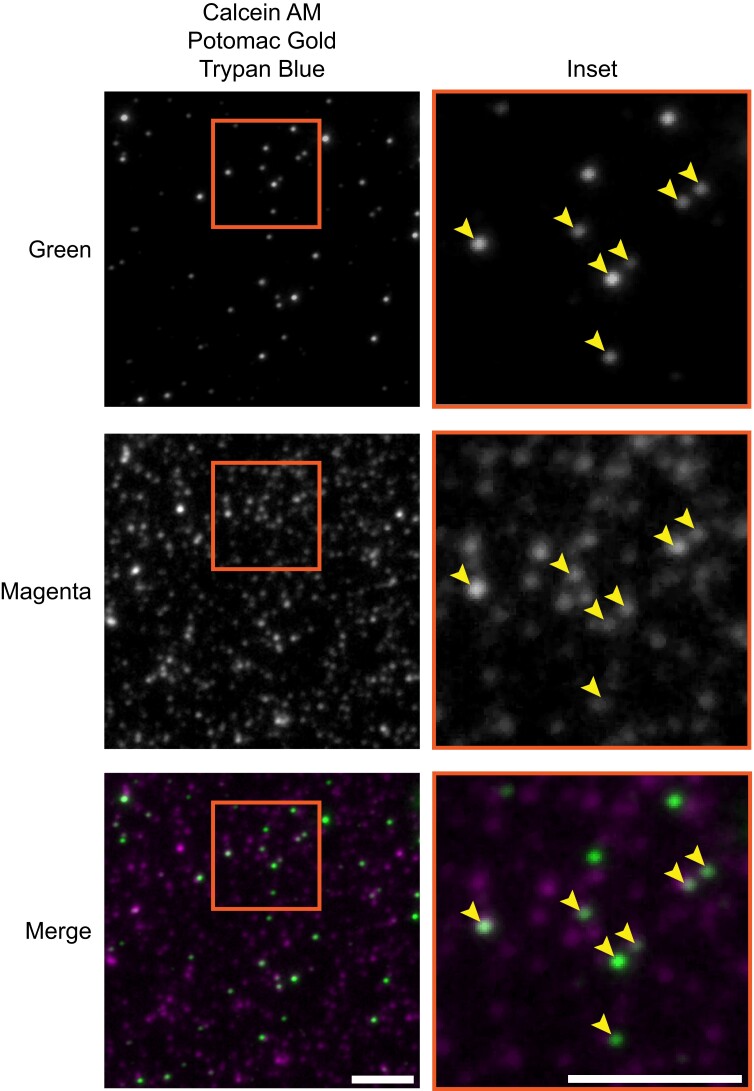
Fluorescent dye labeling of sorghum density-purified EVs. Density-purified sorghum EVs labeled with calcein AM (green) and Potomac Gold (magenta). Images were acquired with BTIRF microscopy, and a median filter of 1 was applied for display. Arrowheads point to EVs labeled with both dyes. Scale bar equals 5 *µ*m.

To determine if these fluorescent foci were membrane-bound EVs, we also labeled the EVs with a lipophilic membrane fluorescent dye and looked for colocalization between the 2 fluorescent signals. We evaluated Potomac Gold, a nonspecific membrane dye known to label membrane-bound organelles in bacteria and mammalian cells (Spahn et al. 2018). Potomac Gold–labeled EV-like objects and fluorescence were retained with the dye trypan blue, which is a dye that will quench unbound background Potomac Gold but not Potomac Gold protected by a membrane (Supplemental Fig. S3). Figure 5 shows colocalization of both the calcein AM green and Potomac Gold–stained objects indicating that the calcein AM green–labeled vesicles are membrane-bound EV-like particles.

### Proteomic analysis

To better understand the protein content of sorghum EVs, as well as to identify potential EV markers, we analyzed sorghum EVs using LC-MS/MS. To remove any contaminating, nonvesicular particles, the crude EV pellets were first purified on a discontinuous iodixanol gradient, as described in Rutter and Innes (2017). Analysis of 3 biological replicates of purified EVs yielded 454 proteins (Supplemental Table S1). From this list, we selected proteins for further analysis that were detected in 2 out of 3 replicates with a *q* ≤ 0.01. The final sorghum EV proteome consisted of 437 proteins (Supplemental Table S2).

**Figure 6.**
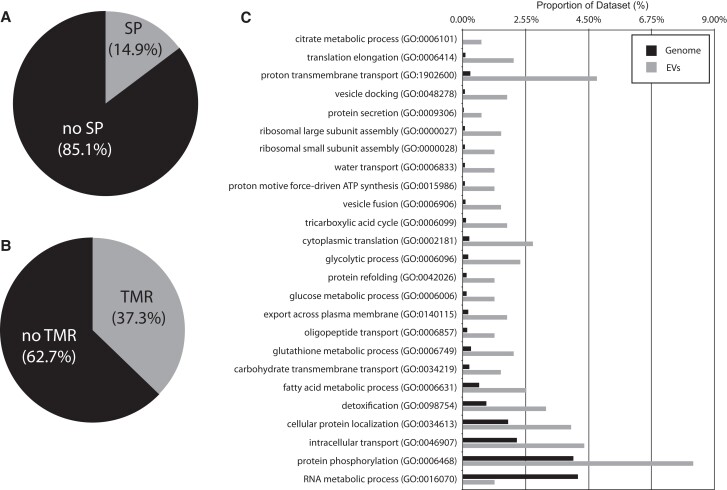
Sorghum EV proteome. Online software predicted 14.9% of sorghum EV proteins possess a SP **A)**. Online software predicts 37.3% of sorghum EV proteins possess at least 1 TMR **B)**. GO term enrichment analysis revealed both enriched and depleted terms in the sorghum EV proteome **C)**.

As membranous compartments involved in the unconventional secretion of proteins, EV proteomes contain a high proportion of membrane proteins and proteins lacking an N-terminal signal peptide (SP) for classical secretion (Rosa-Fernandes et al. 2017). Online software predicted that 14.87% (65/437) of the sorghum EV proteins possessed a SP (Fig. 6A; Teufel et al. 2022), while 37.30% (163/437) of EV proteins were predicted to have at least 1 transmembrane region (TMR; Fig. 6B; Hallgren et al. 2022). This agrees well with data on animal EV proteomes. Around 80% of the top 100 most identified animal EV proteins lack a SP, and 40% possess at least 1 TMR (Rosa-Fernandes et al. 2017). Similarly, 84% of proteins associated with Arabidopsis EVs lacked a SP and 34% possessed at least 1 TMR (Rutter and Innes 2017).

Gene ontology (GO) term enrichment analysis suggests that the sorghum EV proteome is enriched for proteins involved in vesicle docking/fusion, translation/ribosome, transmembrane transport, carbohydrate/amino acid/lipid metabolism, and protein folding (Fig. 6C). Similar GO terms were enriched in EVs from Arabidopsis (Rutter and Innes 2017; He et al. 2021). However, approximately 32% of the sorghum EV proteome was annotated as “uncharacterized,” making it difficult to gain a thorough understanding of the proteome and predict which functions the EVs may possess.

**Figure 7.**
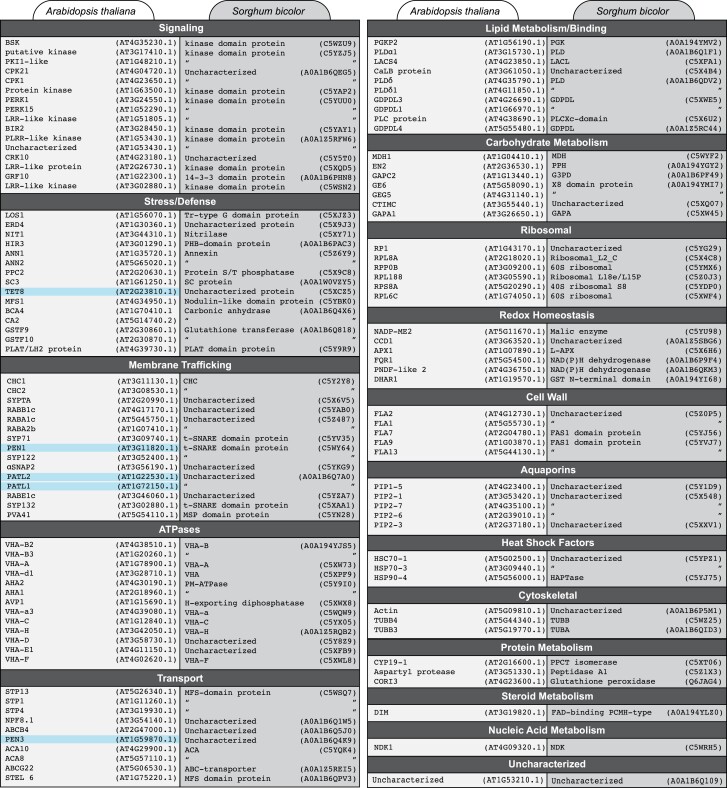
Sorghum orthologs to Arabidopsis EV proteins. Arabidopsis EV proteins were analyzed using NCBI's blastp to identify the closest ortholog (*E* ≤ 1e−50) present in the sorghum EV proteome. Proteins were categorized roughly according to biological function. Proteins commonly identified as Arabidopsis EV markers are highlighted. The presence of a quotation mark indicates that the closest sorghum ortholog is the same as the one listed immediately above.

To gain deeper insight into the sorghum EV proteome and search for similarities between monocot and dicot EVs, we took the Arabidopsis EV proteome published in Rutter and Innes (2017) and searched for the ortholog (*E* ≤ 1e−50) present in the sorghum EV proteome. We found that 60% of Arabidopsis EV proteins had a homolog in the sorghum EV proteome (Fig. 7; Supplemental Table S3). This included homologs to the marker proteins PENETRATION1 (PEN1, AT3G11820.1), PATELLIN 1 (PATL1, AT1G72150.1), the ABC transporter PENETRATION3 (PEN3, AT1G59870.1), and TETRASPANIN8 (TET8, AT2G23810.1), as well as the proposed RNA-binding annexin proteins ANN1 (AT1G35720.1) and ANN2 (AT5G65020.1; Fig. 7). Twenty-nine percent of the uncharacterized sorghum proteins had an annotated ortholog (Fig. 7; Supplemental Table S3). Biological function GO term enrichment of sorghum orthologs suggests a core of proteins enriched for vesicle docking/fusion, transport across membranes, exocytosis, carbohydrate metabolism, and translation (Supplemental Tables S4 and S5). We also identified several orthologs to Arabidopsis stress- and defense-related proteins, suggesting that sorghum EVs may also have a role in immunity (Supplemental Tables S4 and S5).

**Figure 8.**
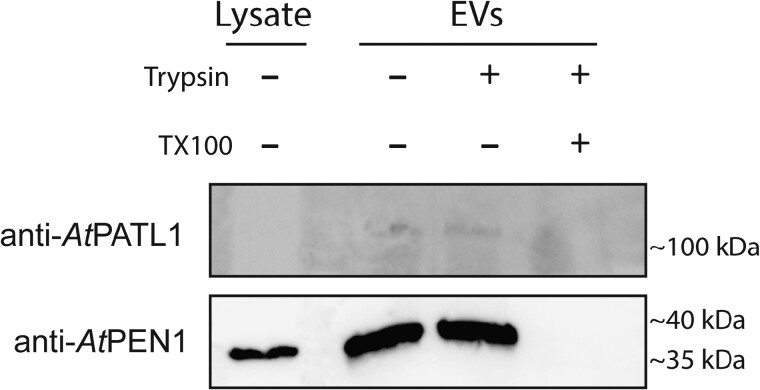
Protease protection assay. Sorghum EVs were treated with buffer, trypsin, and trypsin + TX100 followed by trypsin. Samples were then probed using polyclonal antibodies raised against Arabidopsis EV marker proteins.

To verify our proteomic data and establish tentative EV markers, we conducted a protease protection assay and probed the treated EVs using antibodies raised against common Arabidopsis EV markers PATL1, PEN1, and TET8. In this assay, an EV pellet is divided into 3 samples and treated with buffer, trypsin protease, or Triton X-100 (TX100) detergent followed by trypsin. Proteins protected within the lumen of EVs will remain intact in the presence of trypsin, unless the EV membranes are first solubilized using TX100. When using antibodies specific for Arabidopsis PEN1, we detected bands in buffer and trypsin-treated EV samples (Fig. 8). Anti-PATL1 shows weak reactivity with the trypsin and buffer-treated EV samples. Both the PEN1 and PATL1 bands disappeared when the EVs were pretreated with detergent. The Arabidopsis TET8 antibody was able to detect a strong band that was smaller than the expected size in lysate samples but was unable to detect any bands in EV samples. It is unclear if the band detected in the cell lysate is TET8 or a nonspecific, cross-reacting band. The results suggest that sorghum orthologs of Arabidopsis PEN1 and PATL1 are similar enough to be bound by Arabidopsis-specific antibodies, albeit weakly for PATL1, and that these orthologs are protected within the lumen of membranous compartments. The signal for the band cross-reacting with the PEN1 antibody was particularly strong in both lysate and EV samples. Because sorghum possesses only a single ortholog to PEN1, the t-SNARE C5WY64, the band we detected likely represents the true sorghum PEN1 ortholog (Supplemental Fig. S4). Combined, our data suggest that PEN1 may serve as a useful marker for the identification of both Arabidopsis and sorghum EVs.

## Discussion

Considering research into plant EVs is still in its infancy, not much is known about the proteins associated with EVs from various plant species. Evidence suggests that EVs from Arabidopsis and sunflower (*Helianthus annuus*) are enriched for proteins involved in stress responses and defense, but relatively nothing is known about the protein content of EVs produced by monocots (Regente et al. 2017; Rutter and Innes 2017; Kusch et al. 2023). PEN1 and TET8 were identified as useful marker proteins for the identification and tracking of EVs in Arabidopsis (Rutter and Innes 2017; Cai et al. 2018), but it was uncertain if orthologous proteins can be used to mark monocot EVs. The absence of an appropriate EV marker has been a limitation for further studies in monocots. In this study, we described an improved and optimized protocol involving differential centrifugation for the isolation of EVs from sorghum leaves and have identified EV markers by MS. This proteomic analysis shows that a majority of proteins in the Arabidopsis EV proteome have an ortholog in the sorghum EV proteome, including the commonly used EV markers, PEN1, PATL1, and TET8. Here, we show that PEN1 can be used as an EV marker in the monocot, sorghum. PATL1 also shows promise as a sorghum EV marker if an antibody with higher reactivity was raised; however, our experiments with TET8 were inconclusive. The lack of a TET8-specific band on our western blots of sorghum EV proteins may be caused by poor affinity of the Arabidopsis TET8 antibody to the sorghum TET8 due to sequence divergence. This could be explored in the future by raising an antibody to the sorghum TET8 ortholog. Alternatively, there may be a low, undetectable amount of TET8 in sorghum EVs from plants grown under our growth conditions. Previous work has shown that TET8-positive EVs are induced by a pathogen-induced immune response and exist as a separate vesicle population from PEN1 (He et al. 2021). An innate immune response was not induced in our sorghum plants prior to EV isolation, so it is possible that there were not sufficient TET8 containing EVs to be detected by the antibody. Our study further establishes PEN1 as universal plant EV marker, and when combined with our isolation protocol, it lays the foundation for future studies in sorghum and other monocots.

A major aim of this study was to adapt the Rutter and Innes (2017) Arabidopsis EV isolation protocol for the monocot, sorghum. In general, monocot leaves are more hydrophobic than Arabidopsis leaves, and a critical addition to the sorghum protocol was the snipping of the leaf tips to assist with the vacuum infiltration of the VIB. The second major change was the retention of the buffer used for vAW for subsequent EV isolation. We demonstrated that EVs get released into the buffers during vacuum infiltration, and the yield of EVs can be improved by using both cAW and vAW for downstream processes after infiltration. The addition of the vAW to the EV isolation protocol allowed us to capture vesicles released from the leaves caused by the pressure changes during the vacuum infiltration. The pressure changes cause VIB to enter and exit the apoplastic space via stomata and the cut ends of the vasculature at the ends of the leaves. This process mimics a “washing” of VIB in and out of the leaf with the eventual replacement of all the apoplastic air with VIB, generating a vAW. Future studies in other systems, such as Arabidopsis, would help determine if EV release into vAW is a common outcome.

Cryo-ET analysis of grapefruit PDVs showed similar single- and double-layered vesicles (Garaeva et al. 2021). Many of the vesicles also showed increased electron density, indicating the presence of cargo. Cryo-ET analysis of the sorghum EVs revealed a diverse population of vesicles that were electron dense, multilayered, and a median size of 110 nm. Similar morphological diversity is seen in mammalian-derived vesicles (Emelyanov et al. 2020; Matthies et al. 2020).

In the absence of a suitable EV marker, we attempted to stain sorghum EVs using fluorescent dyes. The dyes that typically have been reported to label vesicles from various plant sources are mainly the lipophilic membrane-staining dyes. It has been reported that PKH26 and PKH67, commonly used lipophilic membrane-staining dyes, can be used to label PDVs isolated from various plant sources. For example, PKH26 has been used to label PDVs from strawberry, grape, grapefruit, ginger, carrot, and lemon juices, whereas PKH67 has been used to label PDVs from cabbage (Mu et al. 2014; Raimondo et al. 2015; Perut et al. 2021; You et al. 2021). Another study reported using dioctadecyl-3,3,3,3-tetramethylindodicarbocyanine (Dil), also a lipophilic membrane dye, to label PDVs isolated from lemon juice (Yang et al. 2020). Cell mask orange (CMO) is also a commonly used lipophilic membrane-binding dye that was reported to label EVs isolated from Arabidopsis (Liu et al. 2020b). These dyes have long aliphatic tails that intercalate into membranes but can also form aggregates that are difficult to distinguish from properly labeled EVs (Melling et al. 2022). Furthermore, a detailed study on the effect of the PKH family of dyes on EVs showed they increase their size. In contrast, luminal dyes, such as carboxyfluorescein succinimidyl ester (CFSE), do not increase the size of EVs, although they will only fluoresce in EVs that can hydrolyze esters and label only a subset of EVs (Dehghani et al. 2020). Here, we used both a luminal, ester-based dye, calcein AM, and a lipophilic dye, Potomac Gold (Spahn et al. 2018). It is unlikely to just bind to the surface of EVs like FM4-64, which has been used previously to stain EVs from sunflowers (Regente et al. 2017), because the addition of trypan blue quenches the fluorescence of Potomac Gold bound to the surface of the coverglass but not from the EVs. This suggests that the lipid bilayer is protecting Potomac Gold from the trypan blue. Lipophilic dyes can also dissociate from membranes, making them unreliable for tracking vesicles (Munter et al. 2018).

Proteins associated with sorghum EVs were similar to those found in other plant EV proteomes. A small percentage of proteins possessed SP, and a larger percentage possessed at least 1 TMR. Sorghum EVs were enriched for proteins involved in vesicle trafficking, the ribosome, transmembrane transport, general metabolism, and protein folding. Similar biological functions were identified for EV proteins from Arabidopsis (Rutter and Innes 2017; He et al. 2021). Moreover, blastp analysis identified orthologs for 60% of Arabidopsis EV proteins in sorghum EVs. Combined, these data suggest that the protein content of plant EVs is partially conserved between Arabidopsis and sorghum. The pathways of EV biogenesis and ultimate function of these vesicles may be similarly conserved. The similarity of the Arabidopsis and sorghum EV proteome suggested that established EV markers for Arabidopsis could be used to identify and validate EVs from sorghum. To this end, we were able to show that antibodies raised against native Arabidopsis EV marker proteins, especially PEN1, were able to detect orthologous proteins in sorghum EVs. Although we were able to detect bands of an expected size using immunoblots, more work is required to confirm detection of a legitimate ortholog. PEN1 belongs to the SYP12 clade of syntaxins that has a conserved function in fungal defense responses. PEN1 does not play a direct role in defense, but rather, it is required for the delivery of defense-related compounds (Liu et al. 2022). Similarly, PEN1 is more likely involved in EV biogenesis rather than be a functional cargo protein. In general, what is considered the cargo of an EV proteome can vary. In this study, we are identifying all proteins that are enriched during a sorghum EV isolation. This includes proteins inside the EVs, its lipid bilayer, the outside surface of the EV, and even proteins that coenrich during the isolation.

## Materials and methods

### Plant material and growth conditions

Sorghum (*S. bicolor*) inbred line BTx623 was used in this study. Seeds were potted using PRO-MIX BK55 growing medium (Premier Tech, LTD, Canada). Between 200 and 250 plants were grown under 16 h days, 27 °C, 350 *µ*mol m^−2^ s^−1^, 65% humidity. Plants were grown for approximately 14 d before harvesting of leaves for the isolation of EVs.

### EV isolation protocol

EVs were isolated from approximately 14-d-old plants following the protocols used by Rutter and Innes (2017) with the following modifications for monocot plants. Before harvesting, 0.5 cm of the leaf tips of each plant was cut to improve vacuum infiltration of buffer. The plants were harvested just above the soil and submerged in water to remove damaged plant material and residual soil. In small groups, the plants were prepared for vacuum infiltration in a 1-L glass beaker using a glass petri dish lid on top of the plant tissue to keep the plant material submerged in VIB (20 mm MES, 2 mm CaCl_2_, and 0.1 m NaCl, pH 6.0). The plants were vacuum infiltrated with VIB using five 20-s cycles at −0.1 MPa with reshuffling of the plants between cycles. After removal of the plants from the 1-L flasks, the remaining buffer was saved as the vAW and was used for subsequent isolation of EVs released into the buffer during the vacuum infiltration process. Small batches of VIB-infiltrated plants were bundled into bouquets with a small strip of parafilm and placed stem side down inside 60-mL syringes. The 60-mL syringes were loaded into 250-mL centrifuge bottles (Nalgene) that were widened to fit a 60-mL syringe and centrifuged at 700 × *g* for 20 min at 4 °C (JA-14 rotor, Beckman Coulter; Avanti J-20 centrifuge, Beckman Coulter) to generate the cAW. The vAW was filtered through a single layer of Miracloth and was combined with the cAW. The combined cAW and vAW solutions were then centrifuged using 29 × 104 mm (50 mL) polypropylene centrifuge tubes (Beckman Coulter, # 357007) at 10,000 × *g* for 1 h at 4 °C (JA-17 rotor, Beckman Coulter; Avanti J-20 centrifuge, Beckman Coulter). The supernatant from this step was decanted into the new tubes and centrifuged at 39,800 × *g* for 2 h at 4 °C (JA-17 rotor, Beckman Coulter; Avanti J-20 centrifuge, Beckman Coulter). The supernatant was then decanted and discarded. The pellet from each tube was resuspended in chilled VIB and pooled together. The resulting solution was then centrifuged again at 39,800 × *g* for 2 h at 4 °C (JA-17 rotor, Avanti J-20 centrifuge, Beckman Coulter). The supernatant was decanted, and the pellet (P40) was resuspended in 100 to 250 *µ*L of 0.2 *µ*m filtered 20 mm Tris-HCl, pH 7.5.

### Density gradient purification

Crude sorghum EVs from the P40 were further purified using a discontinuous iodixanol gradient (OptiPrep, Sigma-Aldrich) following the protocol described by Rutter and Innes (2017). Briefly, 60% aqueous iodixanol stock solution was used to prepare 40% (v/v), 20% (v/v), 10% (v/v), and 5% (v/v) iodixanol dilutions in VIB. Next, the discontinuous density gradient column was prepared by carefully layering 3 mL of 40% solution, 3 mL of 20% solution, 3 mL of 10% solution, and 2 mL of 5% solution in a 14 × 89 mm (13.2 mL) thin-walled ultraclear centrifuge tubes (Beckman Coulter, Cat. # 344059). Approximately 250 *µ*L of the crude EV sample was resuspended in 20 mm Tris-HCl, pH 7.5, and slowly dispensed on top of the column and centrifuged at 100,000 × *g* for 17 h at 4 °C (SW 41 Ti rotor, Beckman Coulter; Optima L-90K, Beckman Coulter). After the ultracentrifugation, 4.5 mL of the solution was discarded from the top of the gradient, and the next 2.1 mL fraction was collected from the gradient, which is the layer spanning the boundary of the 10% and 20% solution and the fraction containing plant EVs (Rutter and Innes 2017). Next, the EV containing fraction was divided into six 350 *µ*L aliquots and dispensed into 1.5-mL polypropylene microfuge tubes (9.5 × 38 mm; Beckman Coulter, Cat. # 357448). The volume in each tube was brought up to 1.5 mL using 20 mm Tris-HCl, pH 7.5. The tubes were then centrifuged at 100,000 × *g* for 1 h at 4 °C (TLA 55 rotor, Beckman Coulter; Optima Max ultracentrifuge, Beckman Coulter). The pellets from the tubes were pooled together in 500 *µ*L of 20 mm Tris-HCl, pH 7.5, buffer and the volume was brought up to 1.5 mL using 20 mm Tris-HCl and centrifuged again at 100,000 × *g* for 1 h at 4 °C (TLA 55 rotor, Beckman Coulter; Optima Max ultracentrifuge, Beckman Coulter). The final pellet containing the GPEV fraction was resuspended in 50 *µ*L of 20 mm Tris-HCl, pH 7.5. Purified EVs were kept at 4 °C and used within 48 h.

### NTA

The concentration and the size distribution of sorghum EVs were determined using NTA as described by Shuler et al. (2020). Briefly, 30 *µ*L of isolated EVs (P40) was added to 970 *µ*L HPLC grade water, and 50 *µ*L of GPEV was added to 450 *µ*L of HPLC grade water. The samples were injected into a Nanosight NS300 (Malvern Panalytical, Malvern, UK) bearing a 532-nm green laser and NS300 FCTP Gasket (Cat. # NTA4137) using a 1 mL sterile BD Plastipak syringe (Becton Dickinson S.A., Madrid, Spain). The following parameters were used for data acquisition: a camera level of 11, a detection threshold of 6 for the P40 fraction and 35 for the GPEV fraction, video duration of 30 s, and 3 technical replicates per sample. NTA software v3.2 was used to analyze the data.

### DLS analysis

DLS and zeta potential analysis were performed with a Litesizer 500 (Anton Paar, GmbH). For both the P40 and GPEV, 30 *µ*L of isolated EVs was resuspended in 970 *µ*L of HPLC water, loaded into a disposable cuvette, and placed in the Litesizer 500. DLS measurements were taken in triplicate.

### Fluorescent labeling and imaging of EVs

EVs isolated in the P40 fraction were stained for 30 min at room temperature with 0.5 *μ*m CellTrace Calcein Green (ex488/em521), AM (Thermo Fisher Scientific), and/or 0.5 *μ*m Potomac Gold (ex561/em594) in 20 mm Tris-HCL, pH 6.0. Potomac Gold was acquired from Luke Lavis (HHMI Janelia). The stained samples were purified using an iodixanol density gradient as described above. The stained EV samples were mounted on 0.1% poly-l-lysine–coated Cellviz dishes (Cat. # D35-10-1.5N), and the background fluorescence of Potomac Gold was quenched using 50 nm trypan blue in HPLC water. Samples were imaged using Borealis total internal reflection fluorescence (BTIRF) or spinning disk confocal microscopy on an Andor Dragonfly 600 microscope (Oxford Instruments) with an Andor Zyla 4.2 PLUS sCMOS camera and a Leica Plan Apo 63× oil immersion TIRF objective (numerical aperture, 1.47). A 2,000 mW, 488 nm excitation laser at 3% and a 521/38 nm bandpass (BP) filter with an exposure time of 500 ms and an averaging of 4 was used for Calcein AM. For Potomac Gold, a 1,000 mW, 561 nm laser at 5% and 594/43 nm BP filter with an exposure time of 500 ms and an averaging of 4 was used.

### TEM analysis

Four hundred mesh carbon-coated copper grids were glow discharged on a Pelco easiGlow (Ted Pella) and placed on 20 *µ*L of EV isolates. The EVs on grids were negatively stained with 2% aqueous uranyl acetate (w/v) and imaged using a Zeiss Libra 120 transmission electron microscope operating at 120 kV. Images were acquired using a Gatan Ultrascan 1000 CCD camera.

### Cryo-ET

The isolation protocol was stopped at the 40,000 × *g* spin, and the pellet was resuspended in 50 *µ*L NaHCO_3_, pH 8.0. Samples were then shipped overnight with cold packs to the Brookhaven National Lab. Cryo-EM grids were prepared on a Vitrobot Mark IV and imaged on a Krios G3i Cryo Transmission Electron Microscope operated at 300 kV. Tilt series were acquired using SerialEM or Thermo Fisher tomography with a nominal magnification of 42,000 and a pixel size of 2.2 Å on a Gatan K3 camera. Tilt series of ±50° were acquired in 2° increments. An energy slit of 20 eV was set on the BioQuantum energy filter for tilt series acquisition. Tilt series were aligned and reconstructed using EMAN2 (Tang et al. 2007) or AreTomo (Zheng et al. 2022). 3D tomograms were loaded into FIJI, average projected and manually analyzed. Single slices of reconstructed tomograms were median filtered for display in figures.

### Correlative imaging by confocal and SEM

Immobilization of EVs on Ibidi Grid-50 coverglasses (Cat. # 10817) was performed using a method adapted from Syga et al. (2018). Coverglasses were sonicated for 30 min in 95% isopropanol (v/v), rinsed 5 times with water, sonicated in 5 N NaOH for 30 min, and rinsed another 5 times with water. A final 30-min sonication in 100% ethanol and 5 times rinses with water was performed prior to air drying the coverglasses with the grid side faced up. HPLC grade water and ethanol were used for all steps, and the glass dish was covered to reduce evaporation during incubations. Cleaned coverglasses were then washed with 100% isopropyl alcohol (IPA), and the excess was baked off at 50 °C. The coverglasses were placed grid side faced up on glass stirring rods taped into a large glass dish. The surface of the coverglasses was flooded with 0.1 N NaOH, incubated at room temperature for 20 min, and rinsed 5 times with water. Excess water was removed and allowed to air dry. Once completely dry, 5% (3-aminopropyl)triethoxysilane (APTES) and 95% IPA (v/v) were applied and incubated for 10 min. The solution was aspirated off, and the excess was removed with 5 times rinses with water. Once completely dry, the grids were incubated for 1 h in 1% glutaraldehyde (v/v) in PBS, pH7.4, that was filtered using a 0.2-*μ*m syringe filter (Fisher 13-100-100). The glutaraldehyde fixative was removed, and 50 *μ*L of P40 EVs prelabeled with calcein AM was applied and incubated for 30 min. To reduce evaporation, moistened kimwipes were placed in the covered glass dish. 0.2 *μ*m filtered 20 mm Tris-HCl, pH 7.5, was added to the coverglasses and then gently removed. Care was taken not to let the surface dry. Additional Tris-HCl was added to the coverglass that was then put into an Attofluor Cell Chamber (Thermo Fisher, Cat #A7816). A larger area was imaged via spinning disk microscopy by acquiring a 3 × 3 tiled *Z* stack in 0.01 nm *Z*-interval steps to account for coverglass levelness. Images were stitched in Imaris Stitcher (Oxford Instruments), and *Z*-maximum intensity projections were made using FIJI (Schindelin et al. 2012). The coverglasses were further processed for SEM by fixing the EVs with 0.2% glutaraldehyde (v/v) in 1× PBS, pH 7.5, for 15 min, washed with HPLC water, and dehydrated in series of solutions with increasing ethanol concentration (Fischer et al. 2012). The coverglasses were then critical point dried using a Tousimis Autosamdri 815A. The coverglasses were mounted on SEM stubs and sputter coated in the ACE600 coater (Leica). SEM image maps were acquired using a Thermo Fisher Apreo VS SEM and the MAPS version 3.9 software (Thermo Fisher Scientific). One or 2 grid squares were selected based on the confocal images, and 7 × 7 image montages were acquired at a setting of 4,096 × 4,096 pixel dimension and a 2-nm pixel size per image. The image montages were stitched, and the confocal images were overlaid and correlated. Image correlation was performed in the Thermo Fisher Maps Offline Viewer V3.

### MS analysis

EV pellets were denatured in a solution of 8 m urea and 100 mm ammonium bicarbonate with a final urea concentration of 1 m. To reduce disulfide bonds, samples were mixed with a final concentration of 10 mm Tris(2-carboxyethyl)phosphine hydrochloride (Cat. # C4706, Sigma-Aldrich) and incubated at 57 °C for 45 min. To alkylate side chains, samples were mixed with a final concentration of 20 mm iodoacetamide (Cat. # I6125, Sigma-Aldrich) and incubated at 21 °C for 1 h in the dark. To digest proteins, 500 ng of trypsin (Cat. # V5113, Promega) was added, and the samples were incubated at 37 °C for 14 h. Following digestion, samples were dried down, resuspended in 0.1% formic acid (v/v), desalted using a zip tip (EMD Millipore), and injected into an Orbitrap Fusion Lumos (Thermo Fisher Scientific) equipped with an Easy NanoLC1200 HPLC (Thermo Fisher Scientific).

The peptides were separated on a 75 *μ*m × 15 cm Acclaim PepMap100 separating column (Thermo Fisher Scientific) downstream of a 2-cm guard column (Thermo Fisher Scientific). A 120 min gradient was run from 4% buffer A (0.1% formic acid in water [v/v]) to 33% buffer B (0.1% formic acid in 80% acetonitrile [v/v]). Peptides were collisionally fragmented using higher-energy collisional dissociation (HCD) mode, and precursor ions were measured in the Orbitrap with a resolution of 120,000 and a spray voltage set at 1.8 kV. Orbitrap MS1 spectra (AGC 4 × 10^5^) were acquired from 400 to 2,000 *m*/*z* followed by data-dependent HCD MS/MS (collision energy 30%, isolation window of 2 Da) for a 3-s cycle time. Unassigned and singly charged ions were rejected through the enabling charge state screening. A dynamic exclusion time of 1 min was used to discriminate against previously selected ions.

### Database search and proteomic analysis

Proteome Discoverer 2.5 (Thermo Fisher Scientific) was used to search the LC-MS/MS data. MS spectra were searched against a *S. bicolor* UniProt database. Parameters for the database search were set as follows: 2 missed tryptic cleavage sites were allowed for trypsin digestion with 10 ppm precursor mass tolerance and 0.05 Da for fragment ion quantification tolerance. Oxidation of methionine was set as a variable modification. Carbamidomethylation (C; +57 Da) was set as a fixed modification. Results were filtered using the Percolator node with a false discovery rate (FDR) of 0.01.

Proteins detected in 2 out of 3 samples with a *q* < 0.01 were selected for further analysis. SP sequences were predicted using SignalP 6.0 (https://services.healthtech.dtu.dk/services/SignalP-6.0/; Teufel et al. 2022). Transmembrane domains were predicted using TMHMM 2.0 (https://dtu.biolib.com/app/DeepTMHMM/run; Hallgren et al. 2022). Orthologs to Arabidopsis EV proteins (those with an *E* ≤ 1e−50) were identified using blastp (https://blast.ncbi.nlm.nih.gov/Blast.cgi?PROGRAM=blastp&PAGE_TYPE=BlastSearch&LINK_LOC=blasthome). GO enrichment was performed using the Panther Classification System provided by the GO Consortium (http://geneontology.org/). Protein alignments were performed using Clustal Omega (https://www.ebi.ac.uk/Tools/msa/clustalo/; Sievers et al. 2011).

### Protease protection assay

The protease protection assay was performed as described in Rutter and Innes (2017). Briefly, EVs were resuspended in 150 mm Tris-HCl, pH 7.8, and divided into 3 samples: A, B, and C. Sample A was treated only with Tris-HCl buffer. Sample B was treated with 1 *μ*g mL^−1^ trypsin (Promega). Sample C was treated with 1% TX100 (v/v) followed by treatment with 1 *μ*g mL^−1^ trypsin (Promega). For the detergent treatment, samples were incubated on ice for 30 min. For the trypsin treatment, samples were incubated in a thermal cycler set at 25 °C for 60 min. All samples were subjected to the same incubation times and temperatures. Following the trypsin treatment step, samples were mixed with 5X SDS buffer and incubated at 95 °C for 5 min. Rabbit polyclonal anti-PEN1 (1:1,000), rabbit polyclonal anti-PATL1 (1:5,000), and rabbit polyclonal anti-TET8 (1:1,000) were used for immunoblotting as previously described (Zand Karimi et al. 2022).

### Accession numbers

Sequence data from this article can be found in the GenBank/EMBL data libraries under accession numbers listed in Supplemental Table S2.

## Supplementary Material

kiad644_Supplementary_Data

## Data Availability

The data underlying this article are available in the article and in its online supplementary material.

## Supplemental data

The following materials are available in the online version of this article.

**Supplemental Figure S1**. Potomac Gold staining of vesicle isolation steps.

**Supplemental Figure S2**. Variation of vesicles seen in cryo-ET images.

**Supplemental Figure S3**. Staining controls for TIRF and spinning disk microscopy.

**Supplemental Figure S4**. Clustal alignment of Arabidopsis PEN1 and sorghum ortholog.

**Supplemental Table S1**. All proteins identified in the sorghum EV proteome by MS.

**Supplemental Table S2**. EV proteins with a *P* ≥ 0.01 in 2 out of 3 replicates.

**Supplemental Table S3**. Sorghum EV proteins with orthologs in Arabidopsis EV proteome.

**Supplemental Table S4**. GO term enrichment of sorghum EV proteins with subsections.

**Supplemental Table S5**. GO term enrichment of sorghum EV proteins without subsections.

## Dive Curated Terms

The following phenotypic, genotypic, and functional terms are of significance to the work described in this paper:

TET8 Gramene: AT2G23810
TET8 Araport: AT2G23810

PATL1 Gramene: AT1G72150
PATL1 Araport: AT1G72150

PEN1 Gramene: AT3G11820
PEN1 Araport: AT3G11820

NTA CHEBI: CHEBI:39054

PEN3 Gramene: AT1G59870
PEN3 Araport: AT1G59870
