# Supplementary material for: The extracellular vesicle proteomes of Sorghum bicolor and Arabidopsis thaliana are partially conserved
Source: Plant Physiol. 2023 Dec 4;194(3):1481–97. doi: 10.1093/plphys/kiad644 (PMC10904328; doi:10.1093/plphys/kiad644)
Supplement: kiad644_Supplementary_Data [file kiad644_supplementary_data.zip › PP2023RA01648D_Supplemental_Figures.pdf]

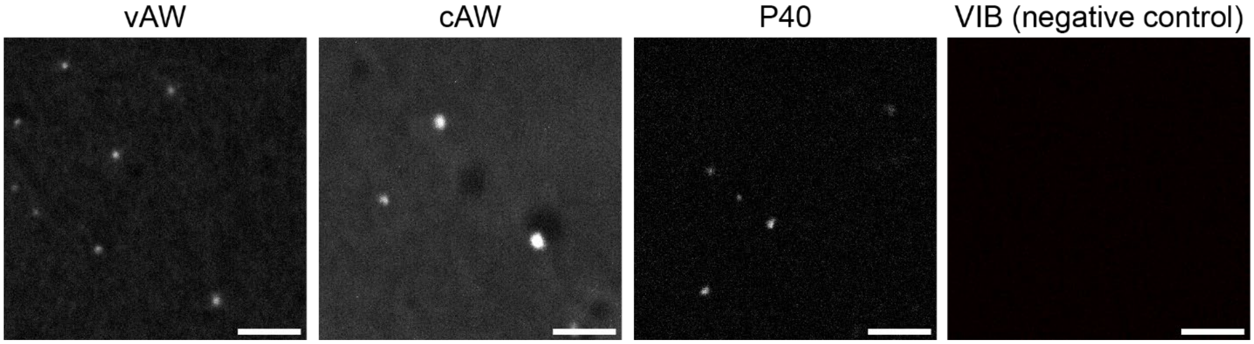

Supplemental Figure S1. Potomac Gold staining of vesicle isolation steps**.** Vacuum apoplastic wash (vAW), centrifugal apoplastic wash (cAW), resuspended pellet from a 40,000 x spin (P40), and vesicle isolation buffer (VIB) negative control all stained with Potomac Gold and imaged via spinning disk. Scale bar equals 10 μm.

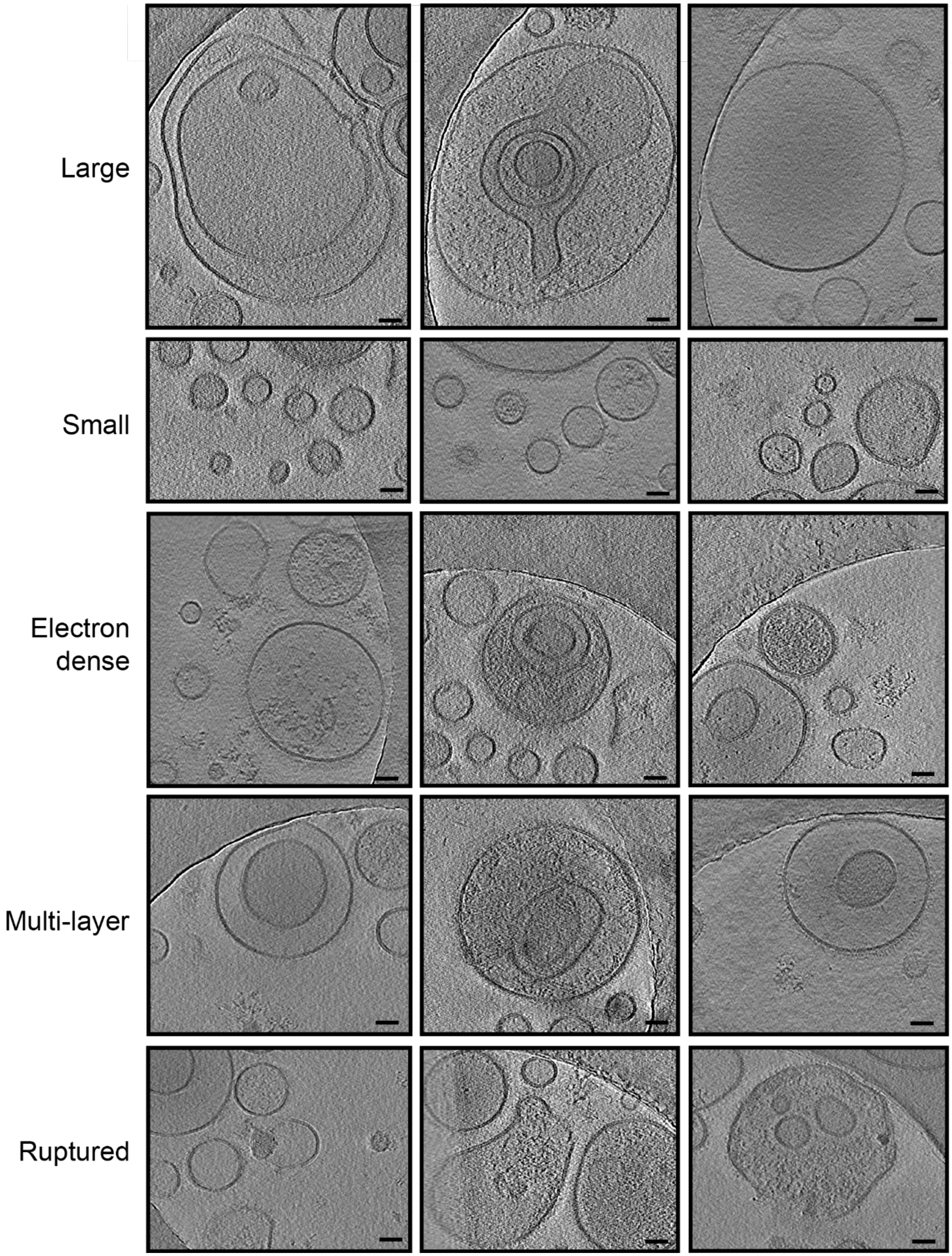
Large
Small
Electron dense
Multi-layer
Ruptured

Supplemental Figure S2. Variation seen in cryo-ET images of sorghum extracellular vesicles. Morphology of sorghum extracellular vesicles (EV) found in cryo-electron tomography (cryo-ET) images. Sorghum EVs were classified as large, small, electron dense, multi-layered, or ruptured. Three representative images (columns) of each EV class are shown and are single slices of reconstructed tomograms. All images were acquired from a single sorghum EV isolation imaged on a Krios G3i. The middle electron dense image shows a large multi-layered EV that is higher density than the surrounding EVs that are also highlighted in first small EV image. Many of the electron dense vesicles also show a hazy fringe around the outside of the lipid membrane. Scale bar equals 50 nm in all images.

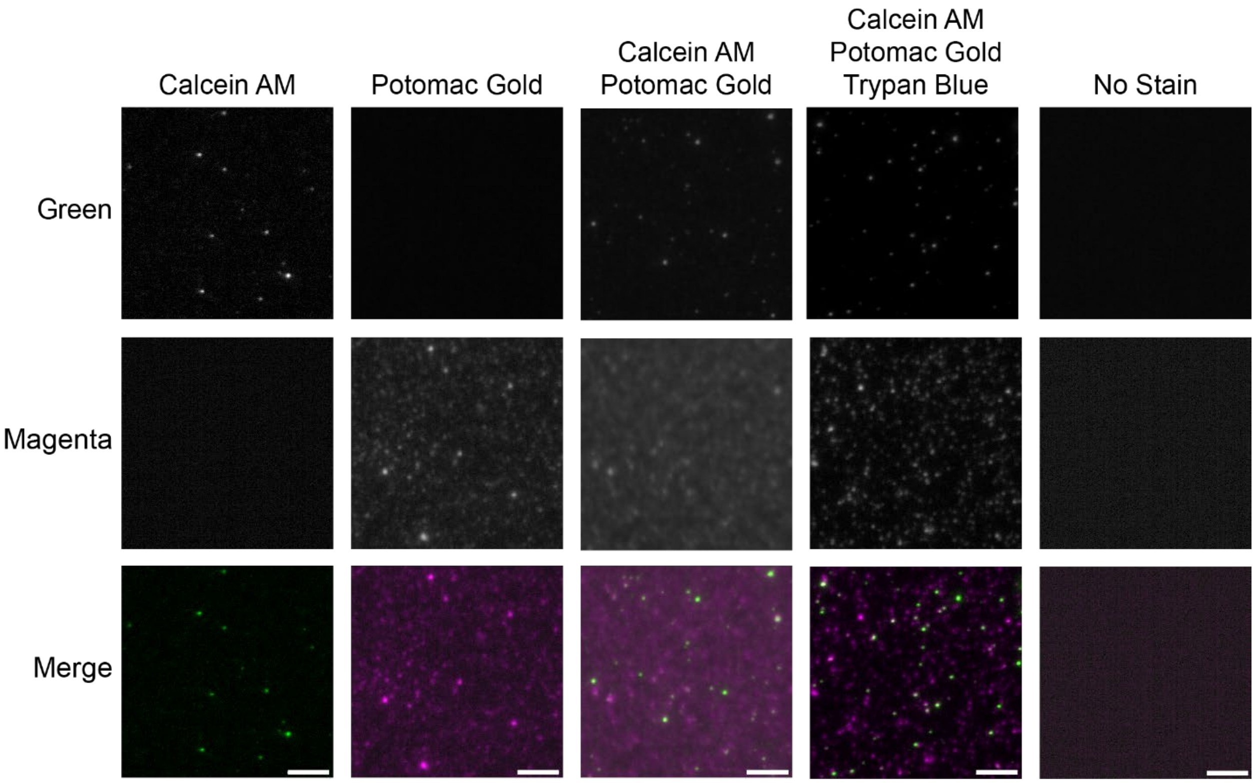

Supplemental Figure S3. Staining controls for total internal fluorescence (TIRF) and spinning disk microscopy. Density purified vesicles stained with Calcein AM, Potomac Gold, both stains combined, both stains with the addition of 50 nM trypan blue and a buffer stain control. Scale bars equal 10 µm.

```
PENETRATION1      MNDLFSSSFSRFRSGEPSPRRDVAGGGDGVQMANPAGSTGGVNLDKFFEDVESVKEELKE   60
C5WY64            MNSLFSSSWKRGGGDDG------DIESGSVEMSAPPGAAAGASLDRFFEDVESIKDELRD   54
                  **.*****:.*  ..:          ...*:*: * *::.*..**:*******:*:**::

PENETRATION1      LDRLNETLSSCHEQSKTLHNAKAVKDLRSKMDGDVGVALKKAKMIKVKLEALDRANAANR   120
C5WY64            LERIQRSLHDGNEAGKSLHDASAVRDLRARMDADVSAAIKKAKVVKLRLESLDRANAANR   114
                  *:*::.:* . :* .*:**:*.**:***::**.**..*:****::*::**:*********

PENETRATION1      SLPGCGPGSSSDRTRTSVLNGLRKKLMDSMDSFNRLRELISSEYRETVQRRYFTVTGENP   180
C5WY64            SVPGCGPGSSTDRTRTSVVAGLRKKLRDSMESFSSLRSRVASEYRDTVARRYFTVTGTQP   174
                  *:********:*******: ****** ***:**. **. ::****:** ******** :*

PENETRATION1      DERTLDRLISTGESERFLQKAIQEQGRGRVLDTINEIQERHDAVKDIEKNLRELHQVFLD   240
C5WY64            DEATLDALAESGEGERFLQRAIAEQGRGQVLGVVAEIQERHGAVAELERSLLELQQVFND   234
                  ** *** * .:**.*****:** *****:**..: ******.** ::*:.* **:*** *

PENETRATION1      MAVLVEHQGAQLDDIESHVGRASSFIRGGTDQLQTARVYQKNTRKWTCIAIIILIIIITV   300
C5WY64            MAVLVAAQGEQLDDIEGNVGRARSFVDRGREQLQVARKHQKSTRKWTCIAILILLVIILV   294
                  *****  ** ******.:**** **:  * :***.** :**.*********:**::** *

PENETRATION1      VVLAVLKPWNNSSGGGGGGGGGGTTGGSQPNSGTPPNPPQARRLLR       346
C5WY64            IVLPIVLQNTKKN---------------------------------       307
                  :** ::   .:..
```

Supplemental Figure S4. Clustal alignment of Arabidopsis PENETRATION 1 (PEN1, AT3G11820) and the sorghum homolog C5WY64.
