# Supplementary material for: The extracellular vesicle proteomes of Sorghum bicolor and Arabidopsis thaliana are partially conserved
Source: Plant Physiol. 2023 Dec 4;194(3):1481–97. doi: 10.1093/plphys/kiad644 (PMC10904328; doi:10.1093/plphys/kiad644)
Supplement: kiad644_Supplementary_Data [file PP2023RA01648DMerged_Decision_Summary_PDF.pdf › PP2023RA01648DMerged_Decision_Summary_PDF.pdf]

Plant Physiology®

Partial conservation of extracellular vesicle proteome between Sorghum bicolor and Arabidopsis thaliana

Timothy Chaya, Aparajita Banerjee, Brian Rutter, Deji Adekanye, Jean Ross, Guobin Hu, Roger Innes, and Jeffrey Caplan

Review Timeline:

Submission Date:	04-Aug-2023
Editorial Decision:	05-Sep-2023
Resubmission Received:	26-Oct-2023
Accepted:	06-Nov-2023

05-Sep-2023

Dr. Jeffrey L. Caplan
University of Delaware
Wilmington, Delaware 19809

RE: Sorghum bicolor extracellular vesicles reveal a conservation between monocot and eudicot extracellular vesicle proteomes

Dear Dr. Jeffrey Caplan:

Thank you for giving us the opportunity to review your manuscript for publication in the Plant Physiology. The reviewers have found the manuscript of interest, but have raised a number of concerns that will require your attention. As these concerns are of some gravity and will entail substantial changes to the manuscript, you are likely to need additional time to address them. I must therefore reject the manuscript in its present form. Nonetheless, if you are able to address the points raised in review, I would welcome your resubmission of a manuscript that incorporates your responses.

In particular, I direct your attention to the following comments for consideration in a resubmitted manuscript.

Correct the typo in the material and methods

Be careful to cite recent reviews

Better discuss the differences or similarities in the density and surface structure of EVs between sorghum and Arabidopsis.

Protease protection assay could be redone presenting clearer results

Please also answer all the other points raised by the reviewers

Please note that any resubmission will be treated as a new entry to the system and is likely to go out for review.

If you do have any queries or concerns, you are welcome to contact me in the first instance.

Thank you again for allowing Plant Physiology to review your work. We look forward to hearing from you soon.

Sincerely,

Plant Physiology Board of Editors

--- Reviewer comments:
Reviewer #1 (Comments for the Author):

The manuscript by Chaya et al. is a major contribution to the recent research focus on extracellular vesicles and their function in plant development and immunity. The novel aspect here is the search for general EV markers plants, with a comparison of dicot and monocot plants.

The authors are experts in the field of plant EV research and have published seminal papers on their isolation and content determination. I find the description of the material and methods clear and comprehensible and based on the authors' experimental experience in the past.

Thus, the manuscript is technically sound; it also contains new useful information, although the data presented here are not very unexpected overall. In fact, there is very little information in the literature on EVs from monocotyledonous plants, which makes the authors' work scientifically relevant.

The manuscript needs to be improved in writing; especially the MM chapter contains many typos.

Minor

L 59: Add more recent reviews?
L104: sentence? And Figure in the Intro?
L109: source ?

I am not sure that the long conclusion, which reads like a discussion, is appropriate, perhaps the conclusion could focus more on the main findings.

Reviewer #2 (Comments for the Author):

This MS by Chaya et al., presents a comprehensive study on the isolation and characterization of extracellular vesicles (EVs) from sorghum leaves, including optimizing the EV isolation protocol, identification of EV markers through mass spectrometry, and analysis of the morphology and size of the isolated EVs. The findings of this study demonstrate the conservation of EV proteomes between sorghum and Arabidopsis, highlighting the potential significance of EVs in intercellular communication and signaling in plants.

However, there are several areas should be paid attention:

Figure 2-5, the authors have provided the solid data concerning the EV from sorghum from the cell biology aspect. Based on the information of this MS and related studies, the density difference and surface structure of EVs could impact their function. The observed difference in density between the different layers of multi-layered EVs suggests that these layers may have different cargo compositions or functions. However, this manuscript does not specifically address whether these characteristics are consistent between monocots and eudicots, which is claimed by the title. Hence, additional investigations or at least further discussions are needed to determine or compare the differences or similarities in the density and surface structure of EVs between these two plant groups to support the authors' hypothesis.

Figure 8, the authors performed protease protection assay to demonstrate PEN1 and PATL1 as universal plant markers, which were protected from Trypsin because they were inside the EVs. However, it's hard to see any WB bands of PATL1 in all samples. Probably the AtPATL1 antibody is not specific to sorghum PATL1. The authors may try again or use other universal EV markers such as TET8 to clarify it.

Line 400, approximately 32% of the sorghum EV proteome was annotated as "uncharacterized". And Line 406, 60% of Arabidopsis EV proteins had a homolog in the sorghum EV proteome including the limited EV markers. It seems that these data do not support the conservations of EV proteomes between monocots and eudicots. The large amounts of new proteins may indicate the interesting functions of sorghum EV and, very likely, the EV proteome shares high similarity with that of maize, as the two species are closely related. Definitely, analysis of a wide range of species is needed to determine the conservation between monocot and eudicot extracellular vesicle proteomes. As this manuscript compared only two species for their EV proteomes, the title of this manuscript is inappropriate.

In addition, "Extracellular vesicles" appeared twice in the title, I suggest the authors to have a concise title

We appreciate the reviews and feedback on our manuscript “*Sorghum bicolor* extracellular vesicles reveal a conservation between monocot and eudicot extracellular vesicle proteomes“, which is now titled, “**Partial conservation of extracellular vesicle proteome between *Sorghum bicolor* and *Arabidopsis thaliana*”.** Below is a point-by-point response regarding the reviewers’ comments.

Reviewer #1 (Comments for the Author):

The manuscript needs to be improved in writing; especially the MM chapter contains many typos.

The manuscript was thoroughly edited and typos have been corrected.

Minor

L 59: Add more recent reviews?

Our introduction cites mainly the primary literature rather than reviews, but we agree that citing reviews also has value. Thank you for the suggestion, and we have added in a number of review citations into the introduction and discussion (Rutter and Innes, 2020; Liu et al., 2021; Wang et al., 2023; Urzi et al., 2021; van der Pol et al., 2012).

L104: sentence? And Figure in the Intro?

The first mention of Figure 1 has been moved to the results section.

L109: source?

We have added in the source of the PRO-MIX BK55 as "Premier Tech, LTD, Canada”.

I am not sure that the long conclusion, which reads like a discussion, is appropriate, perhaps the conclusion could focus more on the main findings.

Plant Physiology research articles have a “discussion” section and we incorrectly titled the last section “conclusions”. We apologize for this oversight. It was written like a discussion section that highlights both the main finding and provides additional insights and perspectives.

Reviewer #2 (Comments for the Author):

This MS by Chaya et al., presents a comprehensive study on the isolation and characterization of extracellular vesicles (EVs) from sorghum leaves, including optimizing the EV isolation protocol, identification of EV markers through mass spectrometry, and analysis of the morphology and size of the isolated EVs. The findings of this study demonstrate the conservation of EV proteomes between sorghum and Arabidopsis, highlighting the potential significance of EVs in intercellular communication and signaling in plants.

We thank the reviewer for recognizing how our manuscript contributes to the plant EV field.

However, there are several areas should be paid attention:

Figure 2-5, the authors have provided the solid data concerning the EV from sorghum from the cell biology aspect. Based on the information of this MS and related studies, the density difference and surface structure of EVs could impact their function. The observed difference in density between the different layers of multi-layered EVs suggests that these layers may have different cargo compositions or functions. However, this manuscript does not specifically address whether these characteristics are consistent between monocots and eudicots, which is claimed by the title.

The title of the paper only claims that the EV proteomes are conserved. However, we have added additional data to show that there are similarities between the surface structure and multi-layering (see below).

Hence, additional investigations or at least further discussions are needed to determine or compare the differences or similarities in the density and surface structure of EVs between these two plant groups to support the authors' hypothesis.

Rather than providing "further discussions", we have provided additional cryo-electron tomography data of *Arabidopsis* extracellular vesicles in Figure 3. These data show that similarities between sorghum and Arabidopsis EVs, including electron dense cargo enclosed by a lipid bilayer and a mixed population of EVs with one and multiple-layers.

Figure 8, the authors performed protease protection assay to demonstrate PEN1 and PATL1 as universal plant markers, which were protected from Trypsin because they were inside the EVs. However, it's hard to see any WB bands of PATL1 in all samples. Probably the AtPATL1 antibody is not specific to sorghum PATL1. The authors may try again or use other universal EV markers such as TET8 to clarify it.

We agree with the reviewer that PATL1 is not a great marker for EVs and the PATL1 data were added as a comparison to the robust data we obtained for PEN1. Additional text was added in to highlight the differences between these two markers. We did not include the TET8 immunoblot because our antibody produces a strong non-specific band in the lysates from both *Arabidopsis* or sorghum. Below is the description in the results section:

> "The *Arabidopsis* TET8 antibody was able to detect a strong band that was smaller than the expected size in lysate samples, but was unable to detect any bands in EV samples. It is unclear if the band detected in the cell lysate is TET8 or a non-specific, cross reacting band."

Line 400, approximately 32% of the sorghum EV proteome was annotated as "uncharacterized". And Line 406, 60% of Arabidopsis EV proteins had a homolog in the sorghum EV proteome including the limited EV markers. It seems that these data do not support the conservations of EV proteomes between monocots and eudicots. The large amounts of new proteins may indicate the interesting functions of sorghum EV and, very likely, the EV proteome shares high similarity with that of maize, as the two species are closely related.

It is a good point that 32% of the proteins are listed as “uncharacterized”, but many of them have homologs in Arabidopsis (Figure 7). We added in text to make this clear. Also, you might infer that if only 60% were found in both EV proteomes then it suggests 40% are unique between them. However, mass spectrometry does not always identify every protein within a sample. Therefore, it is possible that some of the unique proteins in the Arabidopsis or sorghum EV proteomes would be found in both if additional mass spectrometry experiments were conducted. Consequently, we can only state what is conserved rather than what is unique. In-depth studies on unique sorghum proteins is our next avenue of research; however, we believe that it is important to share the isolation protocol, EV characterization and proteome with the community as soon as possible.

Definitely, analysis of a wide range of species is needed to determine the conservation between monocot and eudicot extracellular vesicle proteomes. As this manuscript compared only two species for their EV proteomes, the title of this manuscript is inappropriate.

We thank the reviewer for raising these points. Doing a “wide range of species” is beyond the scope of this manuscript and we hope that the protocol that we developed for over two years will lead to extracellular vesicle studies in other monocots. We also changed our title to “partial conservation” of EV proteomes to reflect that only 60% are conserved and replaced monocot and eudicot with sorghum and Arabidopsis, the two species compared in this study. The new title is: “Partial conservation of extracellular vesicle proteome between Sorghum bicolor and Arabidopsis thaliana”.

In addition, "Extracellular vesicles" appeared twice in the title. I suggest the authors have a concise title.

We simplified our title to only mention extracellular vesicles once. Thank you for the suggestion.

06-Nov-2023
Dr. Jeffrey L. Caplan
University of Delaware
Wilmington, Delaware 19809

MSID: PP2023-RA-01648D
MS TITLE: Partial conservation of extracellular vesicle proteome between Sorghum bicolor and Arabidopsis thaliana

Dear Dr. Jeffrey Caplan:

I am pleased to inform you that your revised manuscript "Partial conservation of extracellular vesicle proteome between Sorghum bicolor and Arabidopsis thaliana" has been accepted for publication in Plant Physiology. Your manuscript will be checked for consistency with journal formatting and image standards by one of our Science Editors, who will contact you shortly. They may have a number of queries and suggestions for you. Please keep in mind that they work to increase the online visibility of your article and its attraction for readers from the widest of backgrounds. You will have the opportunity to review any revisions, and I hope you will work constructively with them to get the most out of your research. You will also have the opportunity to review all changes when you receive the author proofs for your article.

Once validated for publication, your article will be sent to a Science Editor for a final review of your manuscript and source files. After your final files are uploaded, your article will appear in the next available issue and the online Preview version of your article will be posted after your signed Copyright and Checklist forms and final source files are received.

Please be sure to include the following in your manuscript file:
Harvard style citations
List of all author contributions and funding information at the beginning of manuscript after the titles and author list.
List all supplemental materials associated with the article, with titles, at the end of the ms, before the references and acknowledgments section.
Complete Figure Legends for all figures appearing in the article listed before the Literature Cited.

Thank you and congratulations on your Plant Physiology paper!

Sincerely,
Plant Physiology Board of Editors

=====
FIRST AUTHOR PROFILES
=====

We recognize the hard work that goes into being First Author of a Plant Physiology paper by publishing a "First Author Feature" for each First Author alongside your paper.

If the first author(s) would like to submit information for a feature on Facebook and Twitter, fill out the form by clicking on the link below:

Plant Physiology First Author Feature Form: https://forms.office.com/r/ZBCGZfhm8F
If you cannot access the form through the link above, please contact Rachel at rbelsky@aspb.org.

To upload an image of yourself to accompany your profile, please put your photo in the correct journal folder at the link below. You must label your image: Author First Name Last Name Article Title.

First Author Profile Image Dropbox: https://www.dropbox.com/scl/fo/xz029p7qjr2jml2rp2kmq/h?dl=0&rlkey=6fkxcaqmxx35ap152b2osy1fz

=====
IMPORTANT REMINDER: PEER REVIEW REPORTS
=====

If you opted to publish a peer review report along with your article during the original submission process, it will be prepared by the editorial staff and publicly posted with your manuscript, inside the zip file that contains any other supplemental material. As a reminder, the peer review report is a public record of all comments from editors and reviewers, as well as your prior responses, as you received them in the decision letters for each draft of your manuscript. If you agreed to publish this report and have changed your mind, or are not sure if you selected this option, please contact the editorial office as soon as possible before signing the license agreement from our publisher.

--- Reviewer comments:
Reviewer #1 (Comments for the Author):

I appreciate the modification of the thesis with regard to the reviewers' suggestions. Overall, the content of the work was more focussed and the experimental details were expanded based on the reviewers' questions. The change in the title of the ms was also useful and the literature in the introduction was presented in more detail. The extension of the experiment in the direction of cry-ET analysis has significantly strengthened the message of the MS.

Reviewer #2 (Comments for the Author):

The authors have addressed all my concerns and the MS have been improved.
